# Medical students’ knowledge, attitudes, and motivation towards antimicrobial resistance efforts in Eastern Uganda

**DOI:** 10.1371/journal.pone.0314250

**Published:** 2025-02-06

**Authors:** Jonathan Babuya, Daniel Waruingi, Douglas Mungujakisa, Osmas Ahimbisibwe, Victoria Ruth Kako, Faith Aporu, Emmanuel Mugume, Julian Nyamupachitu, Kenedy Kiyimba

**Affiliations:** 1 Faculty of Health Sciences, Busitema University, Mbale, Uganda; 2 Zihi Institute, Nairobi, Kenya; 3 ReAct-Action on Antibiotic Resistance Africa, Lusaka, Zambia; 4 Department of Pharmacology and Therapeutics, Faculty of Health Sciences, Busitema University, Mbale, Uganda; JABSOM: University of Hawai’i at Manoa John A Burns School of Medicine, UNITED STATES OF AMERICA

## Abstract

**Introduction:**

Learning beyond the classroom is important for holistic engagement in antimicrobial resistance(AMR) mitigation. Extracurricular interventions can catalyze multidisciplinary engagement and inspire innovative solutions. This study aimed to determine the knowledge, attitudes, and motivations influencing medical students’ engagement in AMR Club initiatives at Busitema University, Uganda.

**Methodology:**

This was a descriptive cross-sectional study conducted at Busitema University among undergraduate students pursuing Bachelors of Medicine and Surgery, Bachelor of Science in Nursing, and Bachelor of Science in Anesthesia and Critical care. Data collection was performed using a semi-structured, pre-tested questionnaire and administered to the participants The Bloom’s cut-off method was used to analyse the knowledge of the participants, while bivariate analysis was conducted using the chi square test. Multivariable logistic regression analysis was used to identify factors independently associated with students’ engagement in AMR club activities.

**Results:**

Of the 193 study participants, 71.5%(n) demonstrated sufficient knowledge about antimicrobial resistance (AMR), as determined using Bloom’s cutoff categories (≥60% classified as sufficient, <60% as insufficient), with an average knowledge score of 68.18% (SD ±16.12). Additionally, 90% of participants acknowledged the significance of incorporating AMR topics into their curriculum, while 87.5% emphasized the need for AMR training using a One Health approach. The major motivations for students to engage in extracurricular activities addressing AMR were peer influence (n = 42), university support (n = 35), and inspiration drawn from peer mentors’ work (n = 35).

**Conclusion:**

The students demonstrated a high level of knowledge and positive attitudes towards AMR but highlighted the need for further in-depth training. Participation in extra-curricular activities such as involvement in an AMR Club, was found to significantly influence students’ engagement in AMR related interventions.

## Introduction

Antimicrobial Resistance (AMR) has been declared by the World Health Organization as one of the top 10 global health threats [1]. Despite its overarching burden, AMR remains under-prioritized compared to other global health challenges [2]. In 2019, AMR was associated with 4.95 million deaths globally, with 1.27 million directly attributed to resistant infections [3]. In Sub-Saharan Africa, bacterial AMR was associated with 1.05 million deaths and directly caused 250,000 deaths [4]. In Uganda, 7,100 deaths were directly caused by bacterial drug-resistant infections, and 30,700 deaths were associated with drug-resistant infections [4].

Education and awareness play a very critical role in antimicrobial use and shape other health-seeking behaviors that influence AMR [5]. The World Health Organization’s (WHO) Global Action Plan on AMR identifies improving awareness and understanding of AMR as its top priority [6]. It highlights the need to raise awareness and promote behavioral change through various ways such as public communication programs implemented from a One Health realm targeting human health, consumers, animal health, and agricultural practices. It also makes a case for inclusion of AMR into the curricula, and the professional training of health, veterinary, and agriculture sectors to promote a better understanding among professionals.

The Uganda National Action Plan on AMR identifies the promotion of public awareness, training, and education as its first strategic objective. This is pursued through two key strategies: enhancing public awareness and supporting education and training across human, animal, plant, and environmental health sectors through both pre-service and in-service programs. Introducing AMR education early in training and practice equips learners with a different perspective, empowering them to challenge existing norms that hinder antimicrobial stewardship and to become AMR champions within their respective fields. Despite several efforts in Uganda to integrate AMR education into curricula, these initiatives have yet to achieve full success [7].

A study conducted across East African Universities, reported that AMR was not emphasized in the medical curricula [8]. Comparable findings were reported in Ethiopia, Ghana and Zambia among nurses and pharmacy personnel and healthcare students’ in regard to knowledge of Antibiotic use, AMR, and antimicrobial stewardship programs [9]. In Uganda, while the medical students were aware of the concept of AMR, they lacked in-depth knowledge necessary for effective antimicrobial stewardship practices [10]. A research conducted amongst medical, pharmacy and nursing interns also showcased that only a small percentage of participants had good knowledge of AMR and rational prescription practices [11].

AMR clubs are student-led groups established at universities to coordinate and implement activities aimed at combating AMR. These activities include raising awareness through webinars, grand rounds, community outreaches, and celebrating key events such as World Antimicrobial Awareness Week (WAAW). Additionally, the clubs engage in initiatives like AMR runs and quizzes, all designed to enhance understanding of AMR and promote antimicrobial stewardship practices among healthcare students and professionals. All these events are organized under guidance of a senior faculty member who is the patron of the club. These clubs are registered with the university and have an operation structure that is anchored in the club’s constitution. The clubs also have a clear governance structure that comprises of student executives and various supporting committees that meet periodically to plan their activities. There is a clear transitory process to ensure sustainability of the clubs’ activities. Each year there are elections to appoint new executives by the registered members of the club. Each club has a register and any students can also apply to become a member to participate in the club’s activities.

To promote behavior change, and inspire action among healthcare students, engagement should extend beyond traditional classroom settings to include holistic learning that incorporates both curricular and extracurricular activities [12]. From early in their training, tertiary level students should be empowered to engage in AMR. Extracurricular activities such as participation in clubs have been shown to enhance intrinsic motivation, and active participation in efforts to address various global health challenges [13]. To develop supportive frameworks where healthcare students can engage actively and empower communities on AMR, it is best to understand their perspective and drive to engage in such initiatives [14].

Currently, there is a paucity of data from African tertiary institutions on engagement of healthcare students in extracurricular activities on AMR. It is important to have evidence which can be harnessed to promote effective and beneficial extracurricular engagement among healthcare students. This study aimed to determine knowledge and perceptions on AMR among healthcare students and the motivation that influence healthcare students’ participation in AMR extracurricular activities such as clubs.

## Methods and materials

### Study design and setting

This study was a descriptive cross-sectional study employing quantitative data collection method to assess the level of awareness and knowledge of antimicrobial resistance and factors influencing healthcare students’ engagement in AMR club initiatives at Busitema University, Faculty of Health Sciences in Mbale, Eastern Uganda between December, 2023 and February, 2024.

### Study population

These were undergraduate medical students pursuing Bachelor of Medicine and Bachelor of Surgery(MBChB), Bachelor of Science in Nursing(BNS), Bachelor of Science in Anesthesia and Critical care(BNA) at Busitema University.

### Eligibility criteria

The study included undergraduate students at Busitema University, Faculty of Health Sciences who were 18 years of age or older pursuing either Bachelor of Medicine and Bachelor of Surgery, or Bachelor of Science in Anesthesia and Critical Care, or Bachelor of Nursing Science. The students who were not at the Faculty due to other academic programs, such as anesthesia rotations or in their last semester of study were excluded.

### Study variables

#### Dependent variable

The dependent variable was students’ engagement in AMR related activities.

#### Independent variables

These included; socio-demographic characteristics like the age, sex, year of study and course of study, AMR Awareness, Knowledge levels on AMR, and Extracurricular activities engagement.

### Data collection tool and procedures

#### Questionnaire design

We adopted a questionnaire from a study done by Kanyike et al [10], modified it to include questions on attitude and motivating factors for engagement in AMR clubs and then pretested it with 10 students who did not participate in the study. We assessed knowledge using a set of 12 questions marked either correct or incorrect. The percentage pass was then calculated. Blooms cut-off criteria was used to consider those who scored ≥60% to have sufficient knowledge and those <60% to be insufficient. Attitudes were assessed used a set of questions with responses on a likert scale. We uploaded the semi structured questionnaire onto KoBo Toolbox. KoBo Toolbox is an open-source software developed by the Harvard Humanitarian Initiative with support from United Nations agencies, CISCO, and partners to support data management by researchers and humanitarian organizations (https://www.kobotoolbox.org/). The servers are secure and encrypted with strong safe guards and protection against data loss. Participants who consented to the study had the questionnaire link sent to them via a WhatsApp message. The data collection tools were pretested before actual data collection.

### Sample size estimation and sampling technique

The sample size was determined using the formula by Krejcie & Morgan:

$$\text{n}=\left({\text{Z}}^{2}\;\text{x}\;\text{p}\left(1-\text{p}\right)\right)/{\text{d}}^{2}$$

[15].

Where: n = required sample size, *Z* = standard normal deviation at a 95% confidence interval (1.96), *p* = prevalence (0.5 due to no similar study found), *d* = margin of error or degree of accuracy (0.05).

After calculations, *n* = 384.16, rounded to 385 participants. Since the population was less than 10,000, the finite population correction formula was applied. With a population size of 500 students for all the 3 programs, MBChB(n = 288), BNS(n = 112) and BNA(n = 100), the final sample size was determined to be 218 study participants.

A list of all the students at the faculty was obtained, grouped as per the year of study. Random numbers were assigned to the names, they were then filled in a random selector program, that generated a random list of the numbers to which the names were attached. This enabled us to avoid selection bias. The randomly selected students were then approached via a phone call or WhatsApp and given information about the study. Those that consented to participate in the study were recruited and the link to the questionnaire was sent to them. The reasons for engagement in AMR activities were first collected through a pre-study survey among 20 students to populate the questionnaire since we could not find any qualitative study that had been done about these reasons.

### Data analysis and presentation

The data collected was meticulously cleaned to remove duplicates and entries with missing values and analyzed using STATA version 15.0. Categorical data was analyzed using frequencies and percentages, while numerical data was analyzed using measures of Central Tendency and Dispersion. Pie charts were used to visually represent categorical data such as gender distribution, age groups, study programs, and year of study while bar graphs used to illustrate comparisons, such as students’ engagement in extracurricular activities by gender, knowledge levels about AMR across different programs, and motivations for engaging in AMR club activities. Bivariate analysis investigated factors associated with students’ engagement in AMR activities i.e. age groups, study program, year of study, knowledge about AMR, awareness about AMR, and participation in extracurricular activities and presented in tables while multi-variable analysis further examined the significant factors identified in bivariate analysis to determine their combined impact on students’ engagement in AMR activities and presented in figures including pie charts, bar graphs, and other visual aids to succinctly display key findings.

A total of 193 students participated in the study out of the sample size of 218 leading to a response rate of 88.5%. Of the non-respondents, five(5) declined to participate without providing reasons, eight(8) were unavailable due to exchange rotations during data collection, and twelve(12) provided responses with missing values or duplicates, which were excluded from the analysis.

### Ethical consideration

The study was conducted according to the Declaration of Helsinki. Ethical approval was sought and obtained from Busitema University Research Ethics committee under the reference number (BUFHS-2023-104). Written informed consent was obtained from the participants before recruitment into the study. The data collected was kept in a locked folder in the computer which was accessible only to the investigators.

## Results

### Social demographics of the participants

A total of 193 students participated in the study. The majority of respondents were male (58.0%), while females comprised 42.0% of the participants as showed in Table 1. Over half of the respondents (51.3%) were below the age of 25, 40.4% were aged between 25–34, and a small proportion (8.3%) were above 35. The largest group of respondents were enrolled in the Bachelor of Medicine and Bachelor of Surgery program (63.2%). The Bachelor of Nursing Science program had 19.7% of respondents, and the Bachelor of Science in Anesthesia and Critical Care program had 17.1%. The distribution across years of study was relatively even, with the highest percentage in Year 4 (24.4%) and the lowest in Year 2 (17.1%). Year 1, Year 3, and Year 5 had 19.7%, 20.2%, and 18.7% of respondents, respectively(Table 1).

**Table 1:** Showing the demographic statistics of the the study particpants.

	n (%)
**Gender**	
Female	81 (42.0%)
Male	112 (58.0%)
**Age**	
Below 25	99 (51.3%)
25–34	78 (40.4%)
Above 35	16 (8.3%)
**Study program**	
Bachelor of Medicine and Bachelor of Surgery	122 (63.2%)
Bachelor of Nursing Science	38 (19.7%)
Bachelor of Science in Anesthesia and Critical Care	33 (17.1%)
**Year of study**	
Year 1	38 (19.7%)
Year 2	33 (17.1%)
Year 3	39 (20.2%)
Year 4	47 (24.4%)
Year 5	36 (18.7%)

n = 193

### Students’ engagement in extracurricular activities

**Fig 1:**
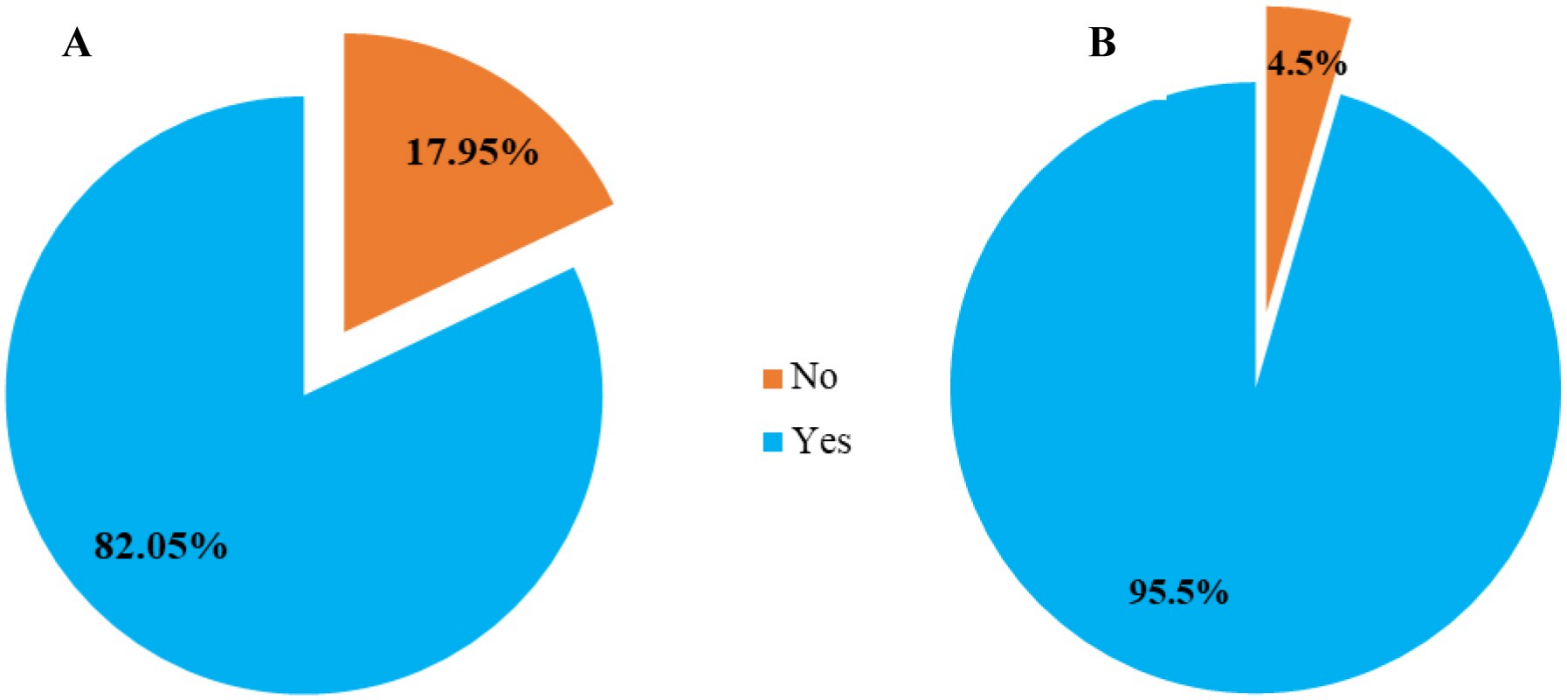
Showing students engagement in extracurricular activities; A; female, B: Male.

Extracurricular activities, in the context of this study, refer to general institutional activities outside the classroom that students participate in. The study found a difference in extracurricular engagement between genders: 82.05% of female students participated in extracurricular activities at the university, compared to 95.5% of male students (Fig 1).

### Students’ engagement in AMR club activities

**Fig 2:**
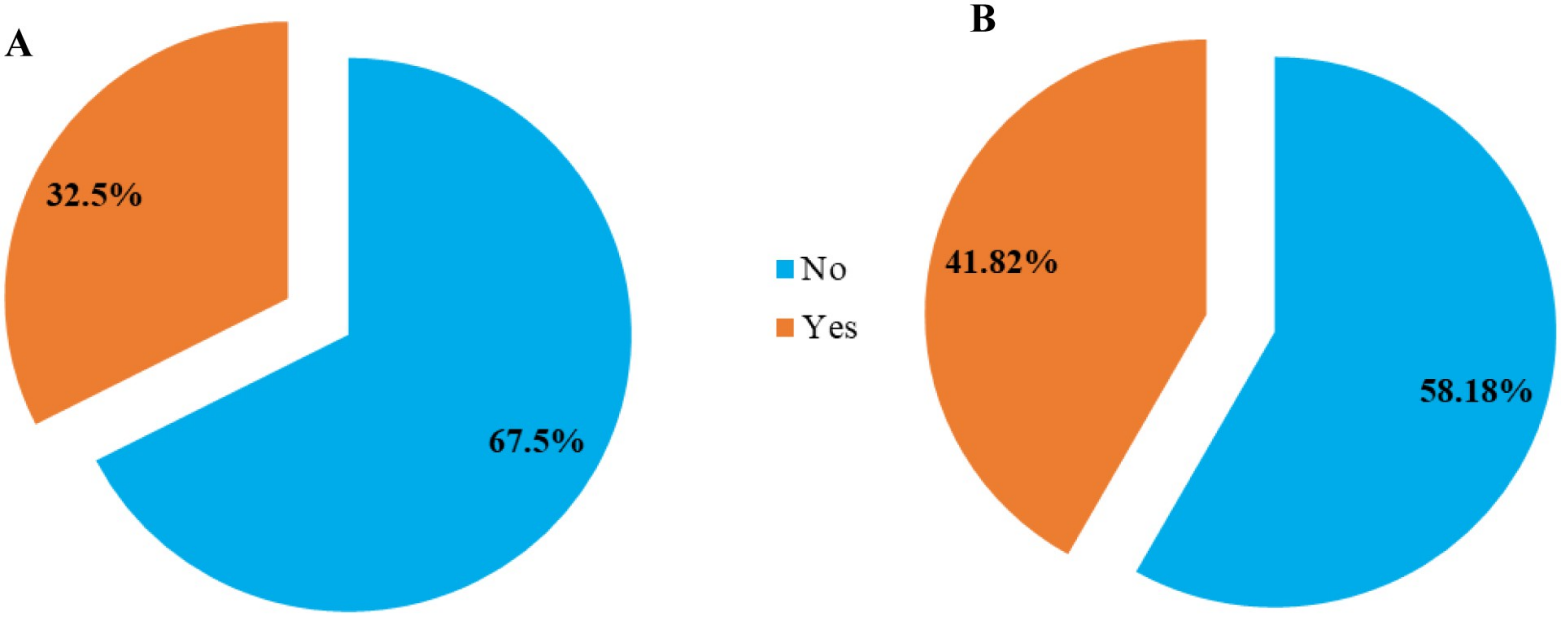
Showing students engagement in AMR activities, A: Female, B: Male.

AMR clubs, which are among extracurricular activities, focus specifically on interventions related to antimicrobial resistance. At Busitema University, 67.5% of female participants reported not having participated in AMR club activities, compared to 58.18% of male students(Fig 2).

### Levels of knowledge about AMR among the students

**Fig 3:**
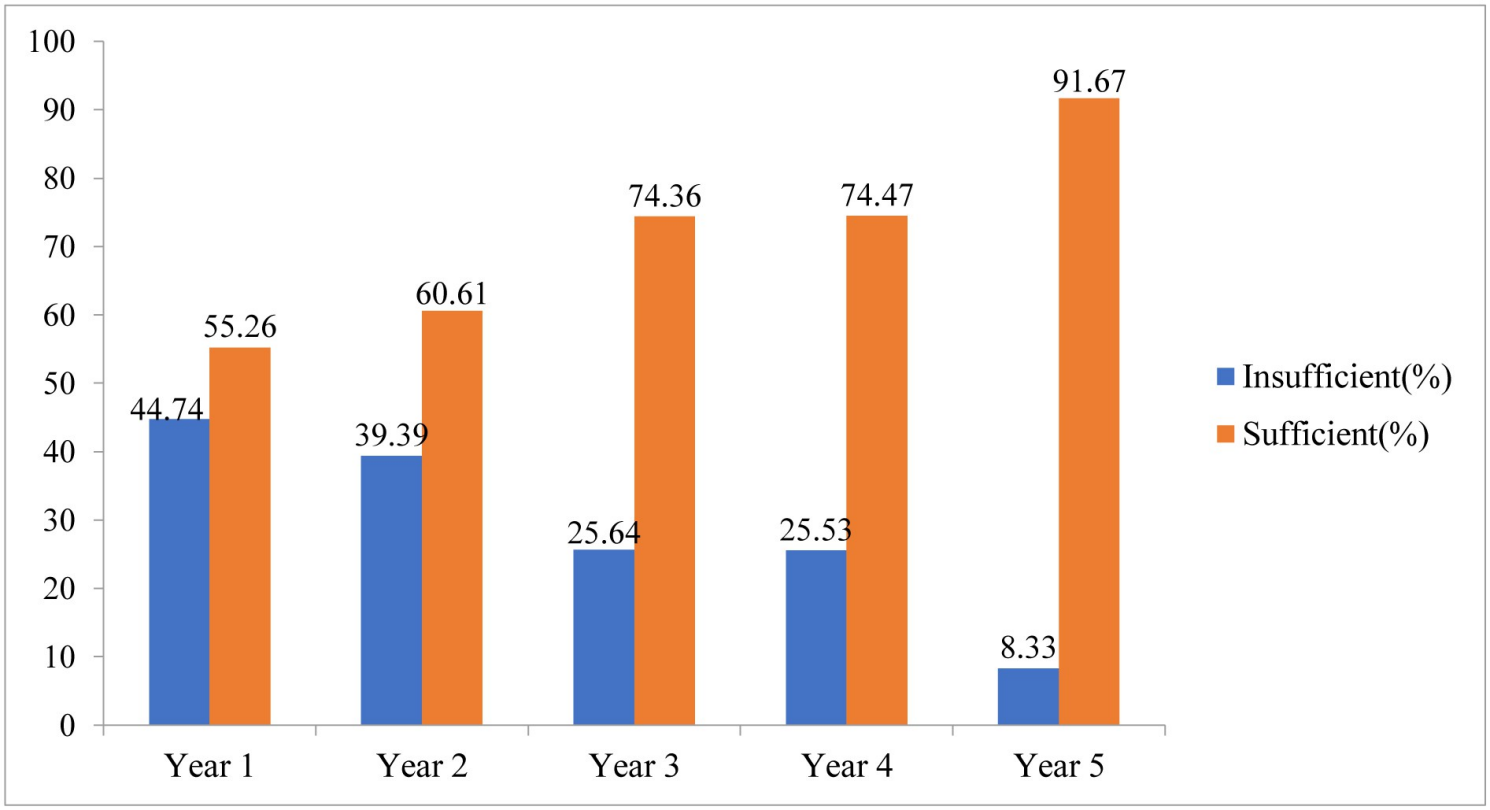
Showing knowledge levels across different years of study.

**Fig 4:**
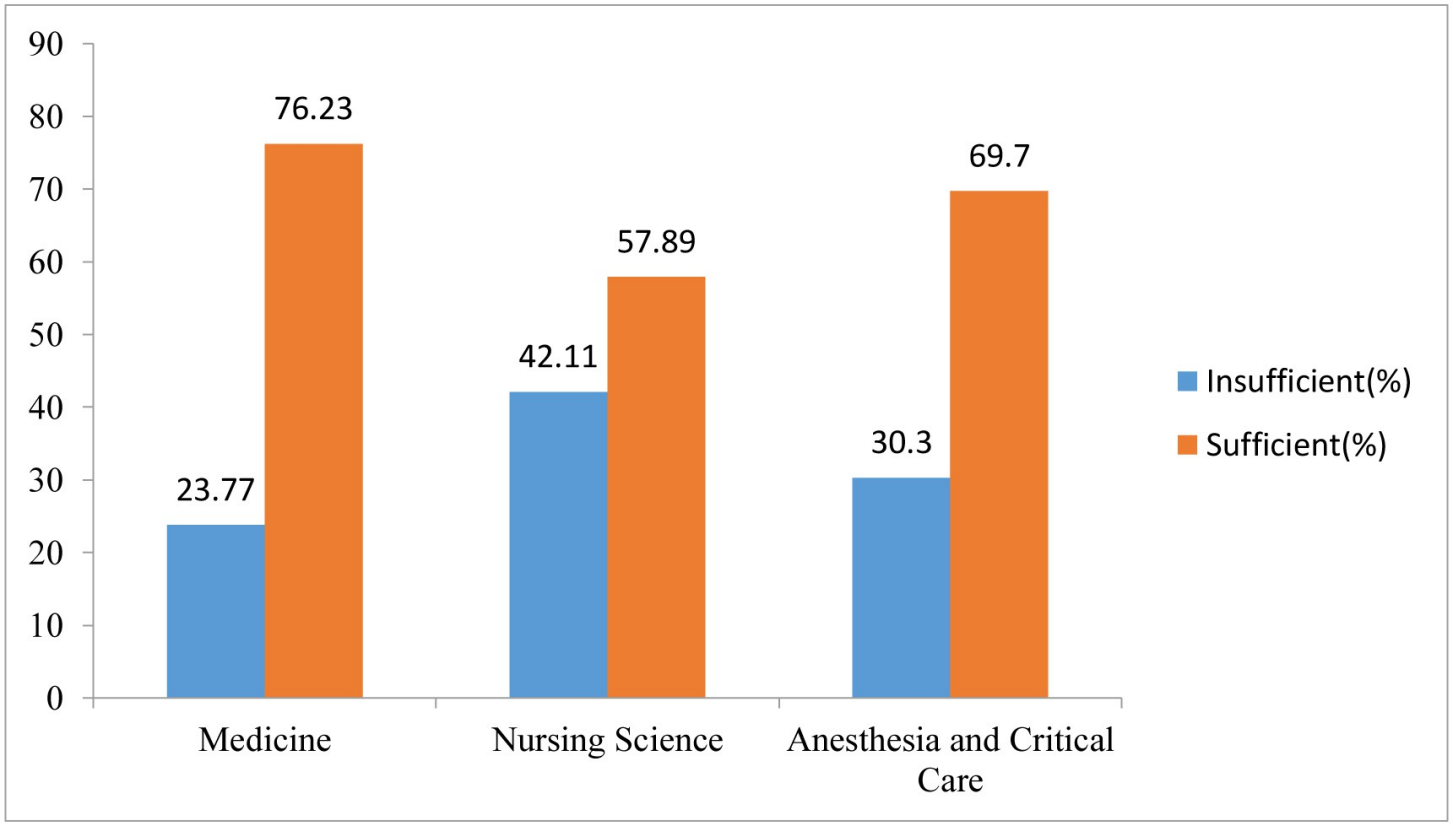
Showing knowledge levels of students in different study programs.

The levels of knowledge were determined through the Bloom Cut off points where students with knowledge scores ≥60% were regarded as having sufficient knowledge, while those with score below 60% were considered having insufficient knowledge about AMR. Overall, 71.5% of the students had sufficient knowledge about AMR. The percentage of students with sufficient AMR knowledge increased with each year of study, starting at 55.26% in Year 1 and rising to 91.67% in Year 5(Fig 3). Across the different programs of study, Bachelor of Medicine and Bachelor of Surgery had the highest percentage, 76.23% of students with sufficient knowledge about AMR which was followed by Bachelor of Science in Anesthesia and Critical Care, 69.7% and Bachelor of Nursing Science at 57.89% (Fig 4).

### Attitudes of students towards AMR

A total of 8.9% (n = 17) of the study participants agreed that agreed that purchasing antibiotics without prescription from pharmacies was acceptable. Over a quarter of the participants (35.3%, n = 68,) agreed to not being at risk of getting an antibiotic-resistant infection as long as they took their antibiotics correctly. Furthermore, an overwhelming majority (83.4%, n = 160) agreed that prescribing broad-spectrum antimicrobials instead of narrower spectrum options contributes to antimicrobial resistance.

Majority of the particpants, 96.4% (n = 186) acknowledged the critical importance of having a strong understanding of AMR in their medical careers. A substantial percentage 87.5% (n = 169) agreed that poor infection control practices by healthcare professionals significantly contribute to the spread of antimicrobial resistance. Similarly, more than 80% of the students (n = 164, 85%) believed that the excessive use of antimicrobials in livestock contributes to the problem of antimicrobial resistance.

**Fig 5:**
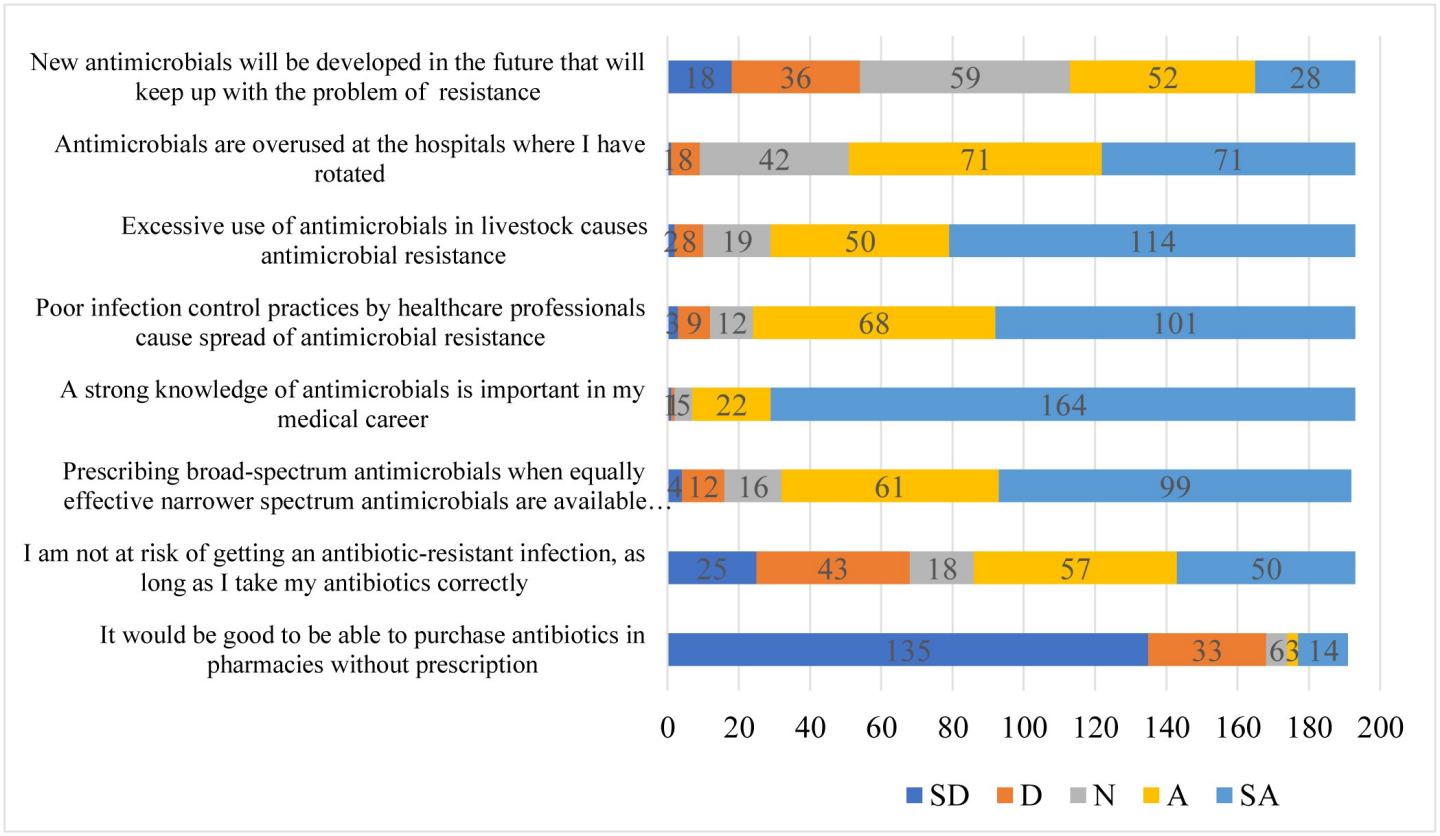
Showing students’ attitudes towards AMR: SD-Strongly disagree, D-Disagree, N- Neutral, A-Agree, SA- Strongly Agree.

Regarding their practical experiences, 73.6% (n = 142) of the students reported observing instances where antimicrobials were overused during their hospital rotations. Looking towards the future, over 40% (41.4%, n = 80) of the participants expressed optimism that new antimicrobial agents capable of addressing resistance issues would likely be developed. These results are summarized in Fig 5.

### Sources of information about AMR

**Fig 6:**
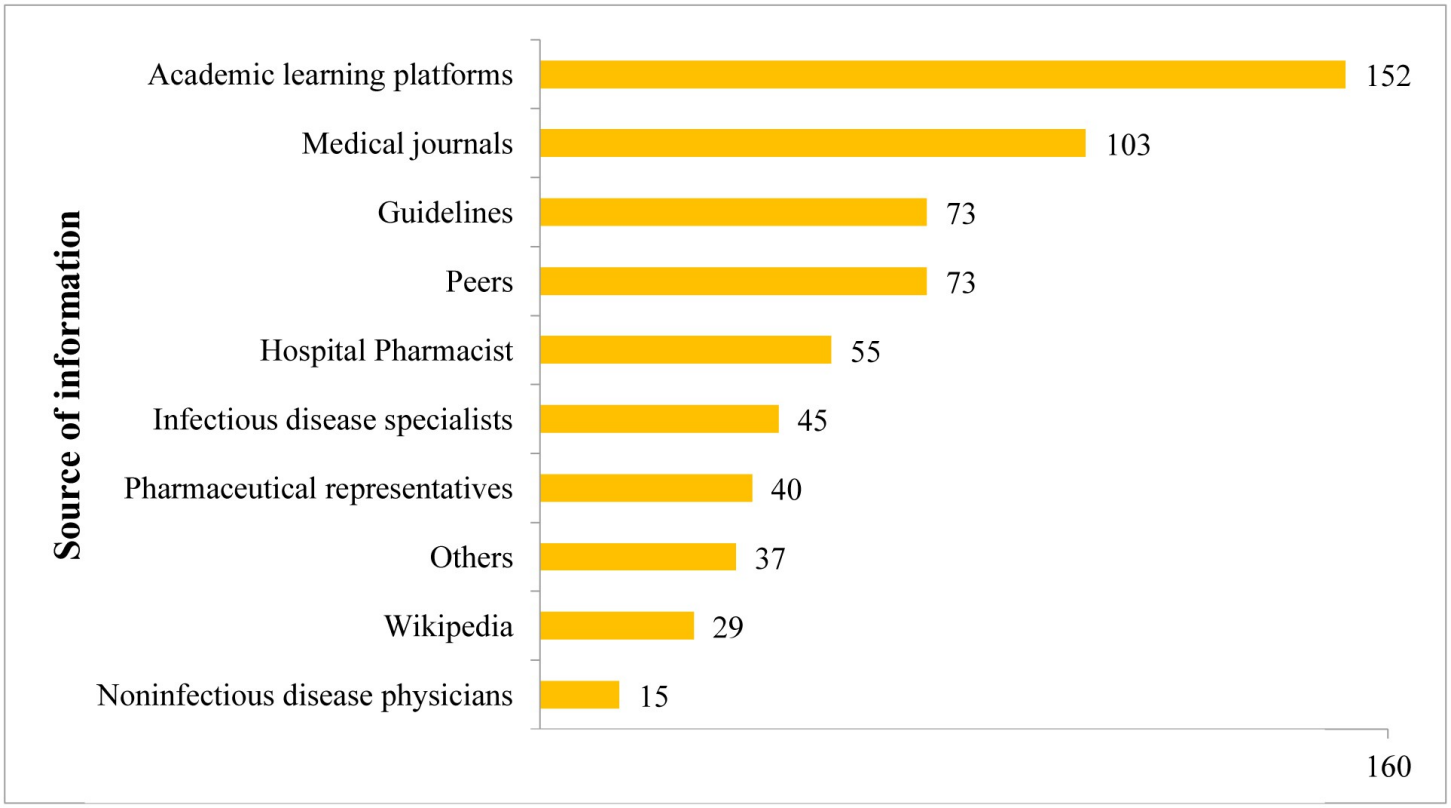
Sources of AMR information used by study participants.

Academic learning platforms were the major sources of information regarding AMR(n = 152). (Fig 6).

### Motivations to students for engaging in AMR club activities

**Fig 7:**
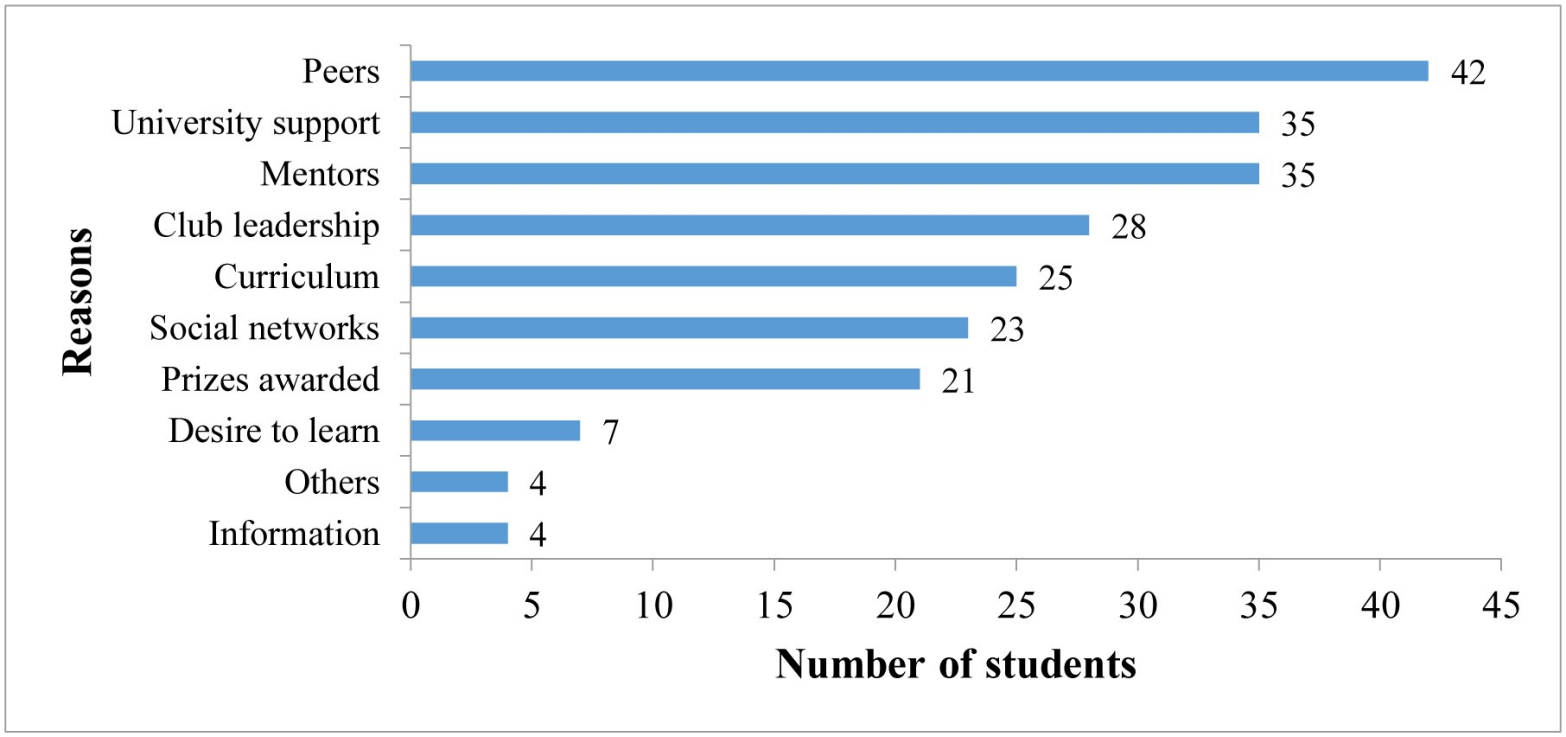
Showing reasons for student engagement in AMR activities.

The largest proportion of students (n = 42) were those motivated by peer to engage in AMR club activities while the least number (n = 4) engaged in AMR activities to get information (Fig 7).

A higher proportion of the students (59%, n = 114) students engaged in extracurricular activities like clubs to create friendships and to exercise and gain physical fitness while the least number of the students engaged in extracurricular activities due to other reasons (n = 5) which included inspiring others and getting knowledge for the future (Table 2).

**Table 2:** Reasons for engaging in extracurricular activities by the study particpants.

	Number of students who selected
	77
To create friendships	114
To exercise and gain physical fitness	114
To develop leadership skills	70
To pass time	51
To contribute to well-being of society	66
To improve my CV	43
To get mentored into my future career	61
To improve job prospects after completing school	41
Other reasons	5

### Factors associated with student participation in AMR club activities

Age, gender and program of study were not significantly associated with student participation in AMR club activities independently. Third year students had 3.12 times higher odds of engaging in AMR club activities than second year students and the result was statistically significant (cOR: 3.12, CI = 1.13–8.66, p = 0.028).

Having sufficient knowledge about AMR had 2.21 times higher odds of engaging in AMR club activities than having insufficient knowledge and the result was statistically significant (cOR: 2.21, CI:1.10–4.42, p = 0.026). Students who were aware about AMR had 3.24 times higher odds of engaging in AMR club activities than those who were not aware and the result was statistically significant (cOR:3.24, CI:1.17–8.96, p = 0.023).

Students engaging in extracurricular activities had 12.55 times higher odds of engaging in AMR club activities than those who were engaging in extracurricular activities and the result was statistically significant (cOR: 12.55, CI: 1.64–96.19, p = 0.015).

However, after adjusting for confounders, student’s engagement in extracurricular activities had 14.45 times higher odds of engaging in AMR club activities than those who did not and the result was statistically significant (AOR: 14.45, CI: 1.84–113.52, p = 0.011) (Table 3).

**Table 3:** Showing bivariate and multivariable analysis for the factors affecting student engagement in AMR.

Independent variables	Categories	Students’ engagement in AMR club activities			
		p-values	Crude Odds Ratios(95%CI)	p-values	Adjusted Odds Ratios(95%CI)
Gender	Male	0.192	1.49(0.82–2.73)		
	Female	*Ref*	*Ref*		
Age(years)	Below 25	0.105	3.00(0.80–11.31)		
	25–34	0.263	2.16(0.56–8.32)		
	Above 35	*Ref*	*Ref*		
Study program	Medicine and Surgery	0.745	1.15(0.51–2.59)		
	Nursing Science	0.509	1.39(0.52–3.67)		
	Anesthesia and Critical Care	*Ref*	*Ref*		
Year of study	Year 1	0.255	1.82(0.65–5.12)	0.401	1.60(0.53–4.80)
	Year 2	*Ref*	*Ref*	*Ref*	*Ref*
	*Year 3	**0.028**	3.12(1.13–8.66)	0.084	2.67(0.88–8.15)
	Year 4	0.095	2.31(0.87–6.19)	0.300	1.78(0.60–5.29)
	Year 5	0.463	1.49(0.51–4.37)	0.973	1.02(0.32–3.24)
Knowledge levels	Insufficient	*Ref*	*Ref*	*Ref*	*Ref*
	*Sufficient	**0.026**	2.21(1.10–4.42)	0.451	1.40(0.58–3.38)
AMR Awareness	Not aware	*Ref*	*Ref*	*Ref*	*Ref*
	*Aware	**0.023**	3.24(1.17–8.96)	0.191	2.34(0.65–8.41)
Extracurricular activities engagement	No	*Ref*	*Ref*	*Ref*	*Ref*
	*Yes	**0.015**	12.55(1.64–96.19)	**0.011**	14.45(1.84–113.52)

## Discussion

The study findings indicate that a higher proportion of the participants (95.5% of the male students, 82% of the female students) participated in extracurricular activities with students in lower academic years exhibiting lower knowledge levels about antimicrobial resistance compared to their fifth-year counterparts. The differences in knowledge levels across the academic years could be attributed to increased exposure to AMR concepts and lectures as students advance in their studies. Additionally, clinical exposure to infectious diseases and AMR increases with study progression as students encounter drug-resistant infections during in clinical rotations particularly in in-patient settings. It is also likely that students in higher years of study have also had more chances to interact with colleagues from other universities and professionals on the subject in various platforms such as conferences, national webinars and student exchanges. These findings are consistent with a previous study, which reported higher knowledge levels among clinical students [10]. Additionally, a study in Ghana showed a sequential increase in AMR knowledge across the years of study, suggesting that students were progressively exposed to more AMR concepts as they advanced in their education [10, 16]. Slightly lower levels of AMR knowledge among students have been reported in various studies across Africa For instance, a study in the Democratic Republic of Congo and Ethiopia found that only 55% of students had sufficient knowledge about AMR, while in Zambia, 70% of students had adequate knowledge levels [9, 17, 18]. These studies suggest a significant knowledge gap among medical students about AMR across the continent and attributed to a diverse factors in different countries [10]. Therefore, there is a critical need for improved education on AMR among medical students in Uganda. Incorporating AMR education into the curriculum can ensure a large number of students are well-informed through a sustainable strategy.

The participants demonstrated a positive attitude towards the rational use of antibiotics, with the majority (87%, n = 168) against the practice of purchasing antibiotics without a prescription. In contrast, a poor attitude was observed among participants in Sudan, where over 59.6% believed it was acceptable to buy medications from a pharmacy without a prescription [19]. Less than 50% of the students (n = 68, 35.3%) agreed to being at risk of getting an antibiotic-resistant infection as long as they took their antibiotics correctly showcasing little understanding on spread of drug-resistant infections, while less than 83.1% that agreed that antimicrobial resistance affects animal health and production and human health-related science students had a positive feeling that inappropriate use of antimicrobials is one of the reasons for the occurrence of antimicrobial resistance [20]. A total of 83.4% (n = 160) agreed that prescribing broad spectrum antimicrobials when narrower spectrum antimicrobials are available increases antimicrobial resistance demonstrating a strong understanding of appropriate prescribing practices. More than 90% of the students(n = 186, 96.4%) agreed that a strong knowledge of antimicrobials is important in the medical career similar to other studies which shows that the overarching recognition on the need to have strong knowledge about AMR as health professionals enabling them to make better decisions for optimal health care delivery [10].

From the findings, 80% of the students (n = 164, 85%) agreed that excessive use of antimicrobials in livestock causes antimicrobial resistance similar to other studies which shows appreciation on the role the one health approach in fight antimicrobial resistance by recognizing other sectors’ importance. About two thirds of the students (n = 142, 73.6%) agreed that antimicrobials were overused at the hospital where they had rotated compared to 36% from a previous study [21]. This shows a better attitude towards recognizing misuse as better stewards of antimicrobials. 41.4% of the students (n = 80, 41.4%) agreed that new antimicrobials will be developed in the future that will keep up with the problem of resistance showcasing poor understanding on the complexity of AMR, and the multifaceted approach required.

Students’ engagement in extracurricular activities was associated with engagement in AMR club activities even after adjusting for other factors. This may be because AMR club activities, being an extracurricular initiative alongside others, were more accessible to students already engaged in similar activities.

Most students engaged in AMR club activities due to influence from peers, university support and mentors while the least engaged to get information and learning. This is mainly because students can easily access their peers and are more inspired and influenced by the actions of people within the same bracket [22]. AMR club activities instill practical skills in addressing AMR like how to communicate effectively about AMR, community organization and mobilization to fight against AMR and good prescription practices learned through webinars and grand rounds. Additionally, the students are exposed to the realities and drivers of AMR at the community level and thus have a higher practical understanding on the topic. The theoretical knowledge learnt is reinforced through experiential learning through activities such as community sensitization, and fosters creativity and innovation among the students. Through their activities, students in the AMR clubs receive invitation to different conferences and symposiums, and are able to expand their network and influence.

Although AMR clubs are important in improving students’ engagement in AMR mitigation activities, it is difficult to incorporate all tertiary students in a club. As such, only a proportion of the students can be reached through this approach. It is therefore important that systemic interventions are put in place that can allow for sustainable understanding and engagement of students within tertiary institutions. One of the sustainable approaches is incorporating AMR into the curricula of different tertiary level courses to ensure that students integrate the knowledge about the fight against AMR. The curriculum could be tailored to the different courses in relation to their clinical practice. This could go a long way in creating AMR stewards who can champion for good prescription, and dispensing practices as well as advocate for rational use of antimicrobials. After completing school, the approach could have a ripple effect creating a new generation of healthcare workers who are more sensitive and informed about AMR mitigation. Interventions towards strengthening peer led initiatives to disseminate information about AMR could prove to be more effective.

### Conclusion

In conclusion, the study reports a sequential increase in knowledge levels on AMR across the years of study amongst the study participants. The study showed that healthcare students appreciated the need and importance of AMR training through a One Health Approach and the importance of good infection prevention and control measures in addressing AMR. Influence from peer, university support and inspiration by peer mentors work were found to be the major reasons behind students’ engagement in AMR related extracurricular activities.

### Recommendations

From our findings, we recommend that, an AMR and AMS course unit should be incorporated into the preservice training of healthcare students so that there is a greater understanding of Antimicrobial Resistance and the topic and potential mitigation efforts. Furthermore, there is need to emphasize on the value of extracurricular engagement in AMR among healthcare students and to leverage on peer support and mentorship to promote holistic learning and effective community engagement in AMR. Lastly, we recommend a broader study incorporating multiple institutions in investigating the drivers and barriers of healthcare students in extracurricular AMR engagements to obtain generalizable results that can inform the country’s AMR policies.

### Limitations

1. The study was done in one university therefore it may be difficult to generalize the findings at a country level.
2. The study population was limited to one faculty which had the highest number of members in the AMR club and did not incorporate other faculties within the university.
3. The use of a cross-sectional design was not sufficient to determine causality among the variables in the study.

## Supporting information

S1 Dataset (XLSX)

S1 Data (PDF)

## References

[1] World Health Organisation. Antimicrobial Resistance: WHO; 2024 [cited 2024 25th October]. https://www.who.int/docs/default-source/antimicrobial-resistance/amr-factsheet.pdf.

[2] Coque TM, Canton R, Perez-Cobas AE, Fernandez-de-Bobadilla MD, Baquero F. Antimicrobial Resistance in the Global Health Network: Known Unknowns and Challenges for Efficient Responses in the 21st Century. Microorganisms. 2023;11(4):1050. Epub 20230417. doi: 10.3390/microorganisms11041050 . 37110473 PMC10144039

[3] Murray CJL, Ikuta KS, Sharara F, Swetschinski L, Robles Aguilar G, Gray A, et al. Global burden of bacterial antimicrobial resistance in 2019: a systematic analysis. The Lancet. 2022;399(10325):629–55. doi: 10.1016/S0140-6736(21)02724-0 35065702 PMC8841637

[4] Sartorius B, Gray AP, Weaver ND, Aguilar GR, Swetschinski LR, Ikuta KS, et al. The burden of bacterial antimicrobial resistance in the WHO African region in 2019: a cross-country systematic analysis. The Lancet Global Health. 2024;12(2):e201–e16. doi: 10.1016/S2214-109X(23)00539-9 38134946 PMC10805005

[5] Fuller W, Kapona O, Aboderin AO, Adeyemo AT, Olatunbosun OI, Gahimbare L, et al. Education and Awareness on Antimicrobial Resistance in the WHO African Region: A Systematic Review. Antibiotics. 2023;12(11):1613. doi: 10.3390/antibiotics12111613 37998815 PMC10669252

[6] Team EE. WHO member states adopt global action plan on antimicrobial resistance. Eurosurveillance. 2015;20(21):21137. 26062562

[7] Waswa J, Kiggundu R, Joshi MP, Mpagi J, Kasujja H, Murungi M, et al. Addressing gaps in AMR awareness in the public: an evidence-based policy brief to guide school curriculum review in Uganda. Frontiers in Public Health. 2023;11:1287523. doi: 10.3389/fpubh.2023.1287523 38074735 PMC10707988

[8] Ampaire L, Muhindo A, Orikiriza P, Mwanga-Amumpaire J, Bebell L, Boum Y. A review of antimicrobial resistance in East Africa. Afr J Lab Med. 2016;5(1):432. doi: 10.4102/ajlm.v5i1.432 28879114 PMC5436405

[9] Tembo N, Mudenda S, Banda M, Chileshe M, Matafwali S. Knowledge, attitudes and practices on antimicrobial resistance among pharmacy personnel and nurses at a tertiary hospital in Ndola, Zambia: implications for antimicrobial stewardship programmes. JAC-Antimicrobial Resistance. 2022;4(5):dlac107. doi: 10.1093/jacamr/dlac107 36226225 PMC9549736

[10] Kanyike AM, Olum R, Kajjimu J, Owembabazi S, Ojilong D, Nassozi DR, et al. Antimicrobial resistance and rational use of medicine: knowledge, perceptions, and training of clinical health professions students in Uganda. Antimicrob Resist Infect Control. 2022;11(1):145. doi: 10.1186/s13756-022-01186-9 36434685 PMC9700951

[11] Nabidda S, Ssennyonjo R, Atwaru J, Kanyike AM, Baryayaka S, Pangholi K, et al. Antimicrobial resistance and rational prescription practices: knowledge, perceptions and confidence of health profession interns in Uganda. JAC-Antimicrobial Resistance. 2023;5(5):dlad105. doi: 10.1093/jacamr/dlad105 37795426 PMC10546811

[12] Munir S, Zaheer M. The role of extra-curricular activities in increasing student engagement. Asian Association of Open Universities Journal. 2021;16(3):241–54.

[13] Achar Fujii RN, Kobayasi R, Claassen Enns S, Zen Tempski P. Medical Students’ Participation in Extracurricular Activities: Motivations, Contributions, and Barriers. A Qualitative Study. Adv Med Educ Pract. 2022;13:1133–41. doi: 10.2147/AMEP.S359047 36176420 PMC9514135

[14] Roulin N, Bangerter A. Extracurricular activities in young applicants’ résumés: What are the motives behind their involvement? International Journal of Psychology. 2013;48(5):871–80. doi: 10.1080/00207594.2012.692793 22823060

[15] Krejcie RV, Morgan DW. Determining sample size for research activities. Educational and Psychological Measurement. 1970;30(3):607–10.

[16] Sefah IA, Akwaboah E, Sarkodie E, Godman B, Meyer JC. Evaluation of Healthcare Students’ Knowledge on Antibiotic Use, Antimicrobial Resistance and Antimicrobial Stewardship Programs and Associated Factors in a Tertiary University in Ghana: Findings and Implications. Antibiotics. 2022;11(12):1679. doi: 10.3390/antibiotics11121679 36551335 PMC9774439

[17] Bogale AA, Amhare AF, Chang J, Bogale HA, Betaw ST, Gebrehiwot NT, et al. Knowledge, attitude, and practice of self-medication with antibiotics among community residents in Addis Ababa, Ethiopia. Expert Review of Anti-infective Therapy. 2019;17(6):459–66. doi: 10.1080/14787210.2019.1620105 31122087

[18] Thriemer K, Katuala Y, Batoko B, Alworonga J-P, Devlieger H, Van Geet C, et al. Antibiotic Prescribing in DR Congo: A Knowledge, Attitude and Practice Survey among Medical Doctors and Students. PLoS ONE. 2013;8(2):e55495. doi: 10.1371/journal.pone.0055495 23441152 PMC3575397

[19] Ibrahim Ahmed MA. Antibacterial Agents: Knowledge and Attitudes of Sudanese College of Medicine Undergraduates. IJCMCR. 2023;30(1). doi: 10.46998/IJCMCR.2023.30.000729

[20] Abunna F, Gebresenbet G, Megersa B. Knowledge, attitude, and practices (KAP) of Univer- sity students regarding antimicrobial use (AMU) and antimicrobial resistance (AMR) in Ethiopia. 2023.

[21] Minen MT, Duquaine D, Marx MA, Weiss D. A Survey of Knowledge, Attitudes, and Beliefs of Medical Students Concerning Antimicrobial Use and Resistance. Microbial Drug Resistance. 2010;16(4):285–9. doi: 10.1089/mdr.2010.0009 20624097

[22] Qureshi MA, Khaskheli A, Qureshi JA, Raza SA, Yousufi SQ. Factors affecting students’ learning performance through collaborative learning and engagement. Interactive Learning Environments. 2023;31(4):2371–91.

