# Supplementary material for: Medical students’ knowledge, attitudes, and motivation towards antimicrobial resistance efforts in Eastern Uganda
Source: PLoS One. 2025 Feb 6;20(2):e0314250. doi: 10.1371/journal.pone.0314250 (PMC11801587; doi:10.1371/journal.pone.0314250)
Supplement: S1 Data — (PDF) [file pone.0314250.s002.pdf]

APPENDIX IV: QUANTITATIVE RESEARCH QUESTIONNAIRE

Title: AWARENESS, KNOWLEDGE AND FACTORS INFLUENCING MEDICAL STUDENTS' ENGAGEMENT IN ANTIMICROBIAL RESISTANCE CLUB INITIATIVES AT BUSITEMA UNIVERSITY.

Section 1: Consent Approval

We are conducting a study to determine the knowledge and perceptions of Antimicrobial Resistance (AMR) among medical students studying at Busitema University, and factors influencing their active engagement in AMR Club Initiatives. This study is aimed at generating evidence to inform strategies that can be used to increase awareness and education on AMR among tertiary level students as well as effective development of systems and structures such as clubs that can improve the active engagement of tertiary level students in AMR.

This study will require you to answer questions provided in the questionnaire, an exercise expected to take 10 – 15 minutes. Participation in this study is voluntary. You can withdraw your participation at any time without any consequences. Participation involves responding to the questionnaire. Your name or any identifiers are not required in the study to ensure the anonymity of the information. All information provided for this study will be treated with utmost confidentiality, and data collected will be used for research purposes only

Question: Do you agree to participate in the study?

Choice 1: Yes (taken to the second option)

Choice 2: No (Exited from the study)

Section 2: Demographic Information

1. **Participant ID**……. (To be filled directly)

2. **Sex**

Choice 1: Female

Choice 2: Male

BUSITEMA UNIVERSITY
FACULTY OF HEALTH SCIENCES
APPROVED VALID UNTIL
★ 02 NOV 2024 ★
RESEARCH & ETHICS COMMITTEE
P. O. BOX 1460, MBALE

3. Age ……. (To be filled directly)

4. Program

A. Bachelor of Medicine and Bachelor of Surgery

B. Bachelor of Science in Anesthesia and Critical Care

C. Bachelor of Nursing Science

5. Year of Study

A. Year 1

B. Year 2

C. Year 3

D. Year 4

E. Year 5

Section 3: Knowledge Section

1. Antibiotic resistance is not a significant problem in my country
   a. Strongly disagree
   b. Disagree
   c. Neutral
   d. Agree
   e. Strongly agree

2. New and stronger antibiotics are being produced by pharmaceutical companies every year
   a. True
   b. False

BUSITEMA UNIVERSITY
FACULTY OF HEALTH SCIENCES
APPROVED VALID UNTIL
★ 02 NOV 2024 ★
RESEARCH & ETHICS COMMITTEE
P. O. BOX 1460, MBALE

[illegible] What is a multidrug-resistant bacterium or a "superbug"?

a. A very pathogenic bacterium capable of causing life-threatening infections.

b. A bacterium that is resistant to several antibiotics

4. Do you know whether your country has a National Action Plan (NAP) for the containment of AMR?

a. Yes

b. No

5. What is the Global Action Plan on Antimicrobial Resistance?

a. A plan, adopted by the World Health Assembly in 2015, containing strategic objectives that indicate what the world should focus on to ensure access and availability to effective antimicrobials for as long as possible.

b. A plan developed by the UN that describes what research areas member states should prioritize to prolong the usefulness of antimicrobials.

c. A plan developed by the global network on AMR aims to inspire diverse stakeholders, policymakers included, to global action on antimicrobial resistance.

6. The national prevalence of Methicillin-resistant Staphylococcus aureus (MSRA) varies from 2% to 50%

A. Yes

B. No

C. Not sure

7. Multidrug resistance Tuberculosis in Uganda is approximately 5-20 percent resistance to isoniazid;

A. Yes

B. No

C. Not sure

8. Do antibiotics speed up the recovery from the common cold and flu?

A. Yes

B. No

C. Not sure

9. Does the frequent use of antibiotics decrease its efficacy?

A. Yes

B. No

C. Not sure

BUSITEMA UNIVERSITY
FACULTY OF HEALTH SCIENCES
APPROVED VALID UNTIL
02 NOV 2024
RESEARCH & ETHICS COMMITTEE
P.O. BOX 1460, MBALE

10. Does inappropriate use of antibiotics put your patients at risk?

A. Yes

B. No

C. Not Sure

11. In poor countries, inadequate access to antibiotics is currently a bigger problem than antibiotic resistance.

a) True
b) False

12. It is necessary to use antibiotics to promote the quick growth of animals and plants

1. Strongly agree
2. Agree
3. Neutral
4. Disagree
5. Strongly Disagree

	Statement	Number of respondents	
1	Are you aware of the term Rational use of medicine?	Yes	No
2	Are you aware of the term essential medicines Lists (EML)?		
3	Are you aware of the P-drug concept?		
4	Can you name the parts of a prescription?		

BUSITEMA UNIVERSITY
FACULTY OF HEALTH SCIENCES
APPROVED VALID UNTIL
02 NOV 2024
RESEARCH & ETHICS COMMITTEE
P. O. BOX 1460, MBALE

5	Are you aware of the STEP (Safety, tolerability, efficacy, price) criteria for the selection of P-drug?		

1. Sources of Information on antimicrobial resistance and rational use of medicines
   A. UpToDate
   B. Medscape
   C. Hospital Pharmacist
   D. Non-infectious disease physicians
   E. Infectious disease specialists
   F. Medical journals
   G. Peers (Other students)
   H. Guidelines by professional organizations
   I. Wikipedia
   J. Pharmaceutical representatives
   K. Others……………………

Section 4: Perceptions of Students about Antimicrobial Resistance

No.	Statement	Strongly Agree	Agree	Neutral	Disagree	Strongly Disagree
1.	It is good to be able to purchase antibiotics in pharmacies without prescription					

BUSITEMA UNIVERSITY
FACULTY OF HEALTH SCIENCES
APPROVED VALID UNTIL
★ 02 NOV 2024 ★
RESEARCH & ETHICS COMMITTEE
P. O. BOX 1460, MBALE

2.	I am not at risk of getting an antibiotic-resistant infection, as long as I take my antibiotics correctly.					
3.	Prescribing broad-spectrum antimicrobials when equally effective narrower spectrum antimicrobials are available increases antimicrobial resistance					
4.	A strong knowledge of antimicrobials is important in my medical career					
5.	Poor infection control practices by healthcare professionals cause spread of antimicrobial resistance					
6.	Excessive use of antimicrobials in livestock causes antimicrobial resistance					

BUSITEMA UNIVERSITY
FACULTY OF HEALTH SCIENCES
APPROVED VALID UNTIL
02 NOV 2024
RESEARCH & ETHICS COMMITTEE
P. O. BOX 1460, MBALE

	Antimicrobials are not used optimally at the hospitals where I have Rotated						
8.	Development of new antimicrobials will be solve the problem of antimicrobial resistance						

Section 5: Active Engagement in AMR Activities

Subsection 1

1. Do you like participating in extracurricular activities?

   a. Yes (directed to Subsection 2)

   b. No (directed to Subsection 3)

Subsection 2

1. What are the main reasons for your engagement in extracurricular activities?

...... (open-ended question)

- To develop talent
- To create friendships
- To exercise and gain physical fitness
- To develop leadership skills
- To pass time
- To contribute to well-being of society
- To improve my CV
- To get mentored into my future career
- To improve prospects of getting a job after completing school
- Other

2. Have you engaged in AMR engagement activities as part of your extra-curricular activities?

   a. Yes(go to the next question)

   b. No(skip the next question)

BUSITEMA UNIVERSITY
FACULTY OF HEALTH SCIENCES
APPROVED VALID UNTIL
02 NOV 2024
RESEARCH & ETHICS COMMITTEE
P. O. BOX 1460, MBALE

3. What are the major reasons that motivated you to actively engage in AMR activities?

- The leadership of the club
- The desire to learn
- Prizes and awards attached to participating
- Peers influence
- Mentorship
- Social Network Engagement
- Curriculum Integration
- University Support
- Accessibility of Information
- Others(specify)…… (open-ended)

4. Have you experienced any challenges at a time when engaging in extracurricular activities?

a) Yes
b) No

5. Could you provide some examples of the challenges

- Lack of facilities
- Lack of support by the university
- Poor participation by colleagues
- Lack of motivation
- Lack of time
- Others(specify)…… (open-ended)

Subsection 3

1. What are some of the reasons that hinder your participation in extracurricular activities like the AMR club?

- Lack of time
- The university does not support these activities
- The club does not engage me
- I need to see impact before I can join the club
- Others(specify)…… (open-ended)

BUSITEMA UNIVERSITY
FACULTY OF HEALTH SCIENCES
APPROVED VALID UNTIL
02 NOV 2024
RESEARCH & ETHICS COMMITTEE
P. O. BOX 1460, MBALE

2. What adjustments do you feel could be made in the way curricular/extracurricular activities such as AMR club are organized to trigger your active participation?
   - Engage experts in the different extracurricular fields
   - Educating students on importance of participating in these activities
   - More time should be set aside for these activities
   - The activities should made mandatory
   - Publicize the club and its activities
   - More funding of the club
   - Meet members more frequently
   - Organize more activities
   - Mobilize more
   - Bring more people on board
   - Others(specify) …… (open-ended)

BUSITEMA UNIVERSITY
FACULTY OF HEALTH SCIENCES
APPROVED VALID UNTIL
02 NOV 2024
RESEARCH & ETHICS COMMITTEE
P. O. BOX 1460, MBALE
